# The Role of Bioelectrical Impedance Analysis in Predicting COVID-19 Outcome

**DOI:** 10.3389/fnut.2022.906659

**Published:** 2022-07-11

**Authors:** Djordje Stevanovic, Vladimir Zdravkovic, Mina Poskurica, Marina Petrovic, Ivan Cekerevac, Nemanja Zdravkovic, Sara Mijailovic, Dusan Todorovic, Ana Divjak, Dunja Bozic, Milos Marinkovic, Aleksandra Jestrovic, Anja Azanjac, Vladimir Miloradovic

**Affiliations:** ^1^Department of Internal Medicine, Faculty of Medical Sciences, University of Kragujevac, Kragujevac, Serbia; ^2^Cardiology Clinic, University Clinical Center Kragujevac, Kragujevac, Serbia; ^3^Pulmonology Clinic, University Clinical Center Kragujevac, Kragujevac, Serbia; ^4^Department of Pathophysiology, Faculty of Medical Sciences, University of Kragujevac, Kragujevac, Serbia; ^5^Department of Medical Statistics and Informatics, Faculty of Medical Sciences, University of Kragujevac, Kragujevac, Serbia; ^6^Ophthalmology Clinic, University Clinical Center Kragujevac, Kragujevac, Serbia; ^7^Department of Ophthalmology, Faculty of Medical Sciences, University of Kragujevac, Kragujevac, Serbia; ^8^Department of Physical Medicine and Rehabilitation, University Clinical Center Kragujevac, Kragujevac, Serbia; ^9^Department of Physical Medicine and Rehabilitation, Faculty of Medical Sciences, University of Kragujevac, Kragujevac, Serbia; ^10^Clinic of Endocrinology, Diabetes Mellitus and Metabolic Diseases, University Clinical Center Kragujevac, Kragujevac, Serbia; ^11^Clinic of Rheumatology and Allergology, University Clinical Center Kragujevac, Kragujevac, Serbia

**Keywords:** body fat percentage, body mass index, COVID-19, obesity, visceral fat

## Abstract

**Background:**

Published data regarding the impact of obesity on COVID-19 outcomes are inconsistent. However, in most studies, body composition was assessed using body mass index (BMI) alone, thus neglecting the presence and distribution of adipose tissue. Therefore, we aimed to investigate the impact of body and visceral fat on COVID-19 outcomes.

**Methods:**

Observational, prospective cohort study included 216 consecutive COVID-19 patients hospitalized at University Clinical Center Kragujevac (Serbia) from October to December 2021. Body composition was assessed using the BMI, body fat percentage (%BF), and visceral fat (VF) *via* bioelectrical impedance analysis (BIA). In addition to anthropometric measurements, variables in the research were socio-demographic and medical history data, as well as admission inflammatory biomarkers. Primary end-points were fatal outcomes and intensive care unit (ICU) admission.

**Results:**

The overall prevalence of obesity was 39.3% according to BMI and 50.9% according to % BF, while 38.4% of patients had very high VF levels. After adjusting odds ratio values for cofounding variables and obesity-related conditions, all three anthropometric parameters were significant predictors of primary end-points. However, we note that % BF and VF, compared to BMI, were stronger predictors of both mortality (aOR 3.353, aOR 3.05, and aOR 2.387, respectively) and ICU admission [adjusted odds ratio (aOR) 7.141, aOR 3.424, and aOR 3.133, respectively].

**Conclusion:**

Obesity is linked with COVID-19 mortality and ICU admission, with BIA measurements being stronger predictors of outcome compared to BMI use alone.

## Introduction

Although most published studies refer to obesity as one of the independent predictors of disease severity and worse outcome in hospitalized COVID-19 patients (1–4), the results regarding mortality are still inconsistent. While some meta-analysis authors observed no significant relationship between obesity and COVID-19 mortality (5, 6), others emphasize such a relationship exists only in younger patients and those with fewer comorbidities (7, 8).

Potential mechanisms by which obesity adversely affects the course of SARS-CoV-2 infection are chronic inflammation and immune response dysregulation, endothelial dysfunction, increased thrombogenic potential, endocrine dysfunction, and the simultaneous presence of other known risk factors (such as cardiovascular disease, metabolic syndrome, and diabetes mellitus) (2, 4, 9).

Given that most of these pathophysiological mechanisms are the effect of adipose tissue (dominantly visceral), the main limitation of published studies is that body composition was assessed solely based on body mass index (BMI), without insight into the presence and distribution of adipose tissue. Moreover, several studies in which abdominal adipose tissue had been assessed using CT scan emphasized the importance of visceral adipose tissue, rather than subcutaneous, on COVID-19 severity and worse outcome (10).

For the reasons stated, it is valuable to examine the impact of body and visceral fat on the course and outcome of the novel coronavirus infection and their correlation with other significant predictors of disease severity, primarily inflammatory biomarkers. In addition, bioelectrical impedance analysis (BIA) measurements could be more precise than BMI in predicting the risk of mortality and worse outcome in hospitalized COVID-19 patients. To the best of the authors’ knowledge, this is the first study regarding BIA measurements and COVID-19 outcomes.

## Materials and Methods

### Study Population

The study was a part of the “COVID-19 admission PREDICTors of OUTCOME” (*COVID-19 PREDICT OUTCOME*) Registry, which was approved by the university’s Clinical Center Kragujevac (Serbia) Ethical Committee.

An observational, prospective cohort study included 216 consecutive COVID-19 patients hospitalized at University Clinical Center Kragujevac (Serbia) from October to December 2021. The patients were followed during the time of hospitalization. Inclusion criteria were adult age (>18 years old) and confirmed SARS-CoV-2 infection. Exclusion criteria were as follows: initial hospitalization at our Center for non-COVID pathology; pregnancy and the early postpartum period; impossibility to perform anthropometric measurements (i.e., poor general condition and severe deformities). In addition, for the reason that only one patient was underweight according to BMI and body fat percentage (%BF), that patient was excluded from further analysis.

### Data Collection

The socio-demographic and medical history data were obtained using the patient’s medical record (Health Informational System, ComTrade, Serbia). Patients were tracked during the hospitalization period, and primary end points were the following: (I) in-hospital mortality, (II) ICU admission, and (III) primary end-point (implying fatal outcome and/or ICU admission) (11).

Within 24 h of admission, a routine laboratory was sampled from peripheral venous blood (complete blood count, biomarkers of inflammation, coagulation parameters, and cardiac biomarkers).

Anthropometric measurements were obtained *via* the BIA method. Using the TANITA BC-543 apparatus (Tanita Corporation, Tokyo, Japan), patients were measured within the first 72 h of hospitalization, according to the manufacturer’s instructions (barefoot, in light clothing, after the morning toilette, and before eating or drinking).

Anthropometric parameters of interest were:

(A) BMI, calculated using the formula: BMI [kg/m^2^] = BM [kg]/BH [m^2^], where BM is body mass expressed in kilograms (with0.1-kg precision), and BH is body height expressed in meters (with0.01 m precision). According to BMI values, patients were categorized as follows: (12). (I) underweight < 18.5 kg/m^2^; (II) normal weight 18.6–24.9 kg/m^2^; (III) overweight 25–29.9 kg/m^2^; (IV) Class 1 obesity 30–34.9 kg/m^2^; (V) Class 2 obesity 35–39.9 kg/m^2^; (VI) Class 3 obesity > 40 kg/m^2^.
(B) % BF, expressed as a percentage of the total mass (with0.1% precision). According to % BF values, regarding age and sex, patients were categorized as follows (13): (I) Low % BF; (II) Normal % BF; (III) High % BF, (IV) Very high % BF (age and sex adjusted cut-off values are presented in Table 1).

**TABLE 1:** Age and sex adjuster cut-off values for body fat percentage (%BF) categories.

Sex	Age (years)	%BF categories			
		Low	Normal	High (overweight)	Very high (obesity)
Female	20–39	<21%	21–33%	33–39.5%	>39.5%
	40–59	<23%	23–34%	34–40%	>40%
	≥60	24%	24–36%	36–41.5%	>41.5%
Male	20–39	<7%	7–20%	20–25%	>25%
	40–59	<10.5%	10.5–22%	22–27.5%	>27.5%
	≥60	<12%	12–25%	25–30%	>30%

*%BF, Body fat percentage.*

(C) Visceral fat (VF) levels, according to which patients were categorized as follows (14): (I) Normal (1–9); (II) High (10–14); (III) Very high (≥15)

### Statistical Analysis

Statistical analysis was performed using the IBM SPSS statistical package version 23 (IBM Corporation, Armonk, NY, United States). The relationship between continuous variables was tested using Spearman’s correlation. Cohen’s kappa coefficient was used in order to measure the level of agreement between different anthropometric measurements in terms of defining obesity. Univariate analysis separately compared anthropometric parameters and other variables with primary end-points. Categorical variables were compared using the χ^2^-test and continuous variables using the Mann-Whitney U test. After identifying the variables associated with end-points, uni- and multivariable binary logistic regression was performed. The strength of the relationship between examined variables and outcome was expressed as odds ratio (OR) belonging to 95% CI for univariate, and as adjusted OR (aOR) belonging to 95% CI for multivariate analysis. *P*-values < 0.05 were considered significant.

## Results

### Cohort Characteristics

Our cohort consisted of 216 adult patients with COVID-19 hospitalized at University Clinical Center Kragujevac (Serbia) from October to December 2021. The patient’s characteristics are presented in Table 2. The median age was 67 years, and the most frequent comorbidities were arterial hypertension, diabetes mellitus, and chronic kidney disease. In our cohort, 16.7% of patients had a fatal outcome, 33.8% required ICU admission, and 35.6% had experienced primary end-point (implying fatal outcome and/or ICU admission).

**TABLE 2:** Cohort characteristics regarding socio-demographic data, comorbidities, and data concerning disease course and outcome.

Cohort characteristics		Percentage (frequency) or median value (with interquartile range)
Sex	Male	63% (136)
	Female	37% (80)
Age (years)		Median: 67.0 (IQR 17.75)
**COMORBIDITIES**		
Arterial hypertension		67.6% (146)
Diabetes mellitus		25.9% (56)
Chronic kidney disease (grade III-V) *		14.4% (31)
Atrial fibrillation		6.9% (15)
Malignancy		6.0% (13)
Previous myocardial infarction		4.2% (9)
Obstructive lung disease **		3.2% (7)
Neurological condition ***		3.2% (7)
Charlson comorbidity index		Median: 3.0 (IQR 2)
**DISEASE COURSE AND OUTCOME**		
Duration between disease onset and hospital admission (days)		Median: 8.0 (IQR 5.0)
Hospital stay (days)		Median: 16.0 (IQR 12.0)
Oxygen support requirement		91.9% (214)
Mortality		16.7% (36)
ICU admission		33.8% (73)
Either primary end-point ****		35.6% (77)

*Chronic kidney disease, estimated glomerular filtration rate below 60 ml/min according to the Cocroft-Gault formula.

**Obstructive lung disease, either chronic obstructive lung disease or bronchial asthma.

***Neurological condition: history of stroke, brain tumor or malformation, vascular disease, dementia of any etiology, etc.

****Either primary end-point—fatal outcome and/or ICU admission.

### Anthropometric Measurements

**FIGURE 1:**
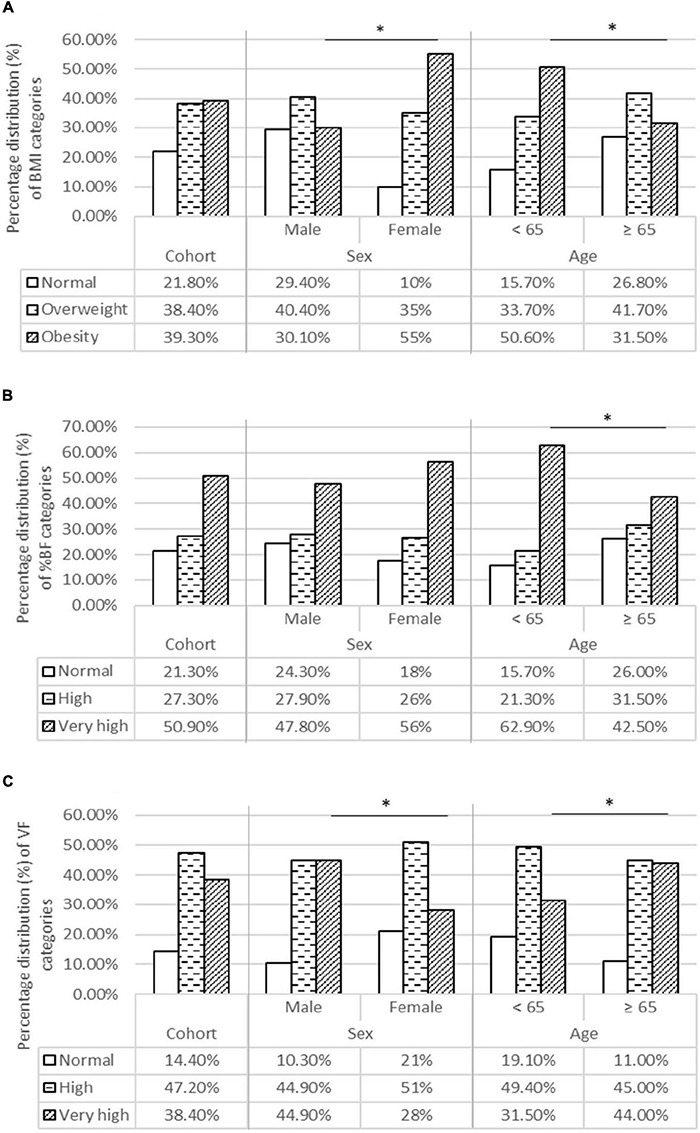
Body composition categories for BMI **(A)**, BF% **(B)**, and VF **(C)** in the entire cohort and for age and sex categories. **(A)** Percentage distribution of BMI categories. **(B)** Percentage distribution of % BF categories. **(C)** Percentage distribution of VF categories. BF %, Body fat percentage; BMI, Body mass index; VF, Visceral fat. *Statistical significance level is taken for “*p*” values below 0,05, using the χ^2^-test.

In our cohort, 39.3% of patients were obese according to BMI, 50.9% had a very high level of % VF, and 57.9% had an excessive level of VF (Figure 1). We noted that older patients had significantly higher values of VF compared to those younger than 65 years, although older patients were less frequently obese according to both BMI and % BF. Regarding sex differences, we observe that women were more frequently obese according to BMI and % BF, whereas men had higher VF levels.

When comparing an agreement between BMI and % BF in terms of defining obesity, we found moderate agreement (*kappa coefficient* 0.543; *p* = 0.045) for three anthropometric categories (eutrophic/overweight/obesity) and good agreement (*kappa coefficient* 0.733; *p* = 0.045) when comparing two groups (obesity/no obesity). However, despite a good agreement between BMI and % BF in defining obesity, 24.5% (*n* = 27) of patients with very high % BF values were categorized as normal-/overweight according to BMI. It is important to point out the high incidence of mortality and ICU admission in this group of patients (24.8 and 55.6%, respectively).

### Obesity and Inflammatory Biomarkers

Upon interpreting associations between predictive laboratory biomarkers on admission [including C-reactive protein (CRP), procalcitonin, interleukin-6 (IL-6), ferritin, lactate-dehydrogenase (LDH), and fibrinogen] and anthropometric parameters, no significant relationship was found regarding BMI. However, patients obese according to BF % had significantly higher serum levels of LDH (median values: 793.5 and 701, respectively; *p* = 0.024) compared to non-obese (including normal and overweight). More interestingly, patients with very high VF levels had significantly higher serum values of CRP (median values: 116.2 and 88.8, respectively; *p* = 0.014) and IL-6 (median values: 88 and 50.4, respectively; *p* = 0.028) compared to those with normal/high VF levels. Statistical significance for other biomarkers was not found (Supplementary Table 1).

### Body Composition and Primary End-Points

Table 3 shows crude and adjusted OR for different anthropometric measurements in regard to predicting primary end-points. Initially, % BF and VF levels were significant predictors of mortality, while BMI, although borderline, lacked statistical significance. However, after adjusting OR for age, sex, days from symptom onset, and obesity-related comorbidities (diabetes mellitus), all three anthropometric measurements were statistically significant predictors of mortality, with % BF and VF being stronger predictors compared to BMI.

**TABLE 3:** Crude and adjusted OR (with 95% CI and “*p*” values) for different somatometric measurement methods in regard to primary end-points occurrence (mortality, ICU admission, and either primary end-point).

Somatometric measurement method	Body composition categories	End-point occurrence (%)	Crude OR (95% CI)	*P*	Adjusted OR* (95% CI)	*P*
**Mortality**						
BMI	Non-obese	13%	1		1	
	Obese	22.4%	1.930 (0.939–3.971)	0.075	**2.387** (1.067–5.337)	0.034
BF%	Non-obese	10.4%	1		1	
	Obese	22.7%	**2.540** (1.179–5.470)	0.017	**3.353** (1.471–6.642)	0.004
VF	Normal/High	10.5%	1		1	
	Very high	22.6%	**3.066** (1.466–6.411)	0.003	**3.050** (1.407–6.609)	0.005
**Critical form**						
BMI	Non-obese	22.1%	1		1	
	Obese	51.8%	**3.775** (2.087–6.827)	0.001	**3.113** (1.663–5.825)	0.001
BF%	Non-obese	13.2%	1		1	
	Obese	53.6%	**7.602** (3.868–14.960)	0.001	**7.141** (3.538–14.413)	0.001
VF	Normal/High	26.3%	1		1	
	Very high	56.8%	**2.364** (1.325–4.219)	0.004	**3.424** (1.781–6.581)	0.001
**Composite outcome**						
BMI	Non-obese	25.2%	1		1	
	Obese	51.8%	**3.187** (1.784–5.693)	0.001	**2.769** (1.495–5.125)	0.001
BF%	Non-obese	16%	1		1	
	Obese	54.5%	**6.282** (3.312–11.918)	0.001	**6.085** (3.121–11.862)	0.001
VF	Normal/High	27.8%	1		1	
	Very high	48.2%	**2.414** (1.360–4.284)	0.003	**3.208** (1.705–6.035)	0.001

*% BF, Body fat percentage; BMI, Body mass index; CI, Confidence interval; ICU, Intensive care unit; OR, odds ratio; VF, Visceral fat.*

*OR values with a statistical significance level of <0.05 are presented in bold.*

*OR was adjusted for age, sex, days from disease onset, and diabetes mellitus.

In predicting ICU admission and the development of either primary end-point, all three anthropometric measurements were significant predictors before and after adjustment. Similar to mortality prediction, % BF and VF had higher aOR compared to BMI.

The impact of socio-demographic characteristics and comorbidities on primary end-point occurrences are presented in Supplementary Table 2.

## Discussion

The research was conducted on 216 patients with COVID-19 consecutively hospitalized at our Center between October and December 2021, in a period of the presumed predominance of SARS-CoV-2 delta variant in our country. The majority of hospitalized COVID-19 patients in our cohort had disturbed body composition, with only two out of ten hospitalized patients having normal BMI and % BF, and 14.4% of patients having normal VF levels. The shown disturbances of body composition in hospitalized COVID-19 patients are not unexpected. First, obesity is a globally raging pandemic whose consequences are also noticeable in Serbia. According to a WHO report from 2013, 58.6% of the adult population in Serbia were overweight or obese, and, according to a model at the time, the predicament was that the obesity prevalence in 2020 would be 44% in adult men and 31% in adult women (15). Second, several studies have demonstrated that obesity is a significant risk factor for hospital admission (3, 6, 16), therefore it is somewhat expected to have a high prevalence of obesity among hospitalized COVID-19 patients. We must note that only one patient (man, 70 years old) was underweight according to BMI and % BF; therefore, he was neglected in further analysis. Although some studies have shown an increased risk for death and the need for mechanical ventilation in underweight patients (17).

Regarding age categories, patients younger than 65 years had a higher prevalence of obesity according to BMI and % BF measurements. In contrast, older patients had significantly higher VF levels. We accentuate that the high VF levels are associated with numerous health disorders and general mortality (18, 19), along with worse outcomes and death in patients with COVID-19 (10, 20–23). Therefore, excessive VF levels could be one of the links associated with increased mortality and severity in older patients with COVID-19, among others (7, 16, 24, 25). Moreover, some studies advocate obesity as a risk factor for COVID-19 mortality and severity dominantly for younger patients, with weaker or no impact at all in older patients (5, 26, 27). Perhaps this could be a misconclusion, for cited studies have used BMI alone as a tool for accessing obesity, neglecting the significance of VF (7, 16, 24, 25).

In the initial analysis, only % BF and VF level were significant predictors of mortality, while BMI, although borderline, lacked statistical significance (Table 3). However, after adjusting OR for age, sex, days from disease onset, and obesity-related comorbidities (diabetes mellitus), all three anthropometric methods were significant predictors of mortality, with both % BF and VF having higher aOR values compared to BMI (aOR 3.353, aOR 3.05, and aOR 2.387, respectively). Results regarding ICU admission and experiencing either primary end-point were more concordant, where all three anthropometric measurement methods had significant predictive importance, with % BF (aOR 7.411 and 6.085, respectively) and VF (aOR 3.424 and 3.208, respectively) again having higher aOR values compared to BMI (aOR 3.113 and 2.769, respectively).

We must note that comorbidities selection for OR adjustment was arbitrary, and diabetes mellitus was chosen as a known obesity-related condition. Furthermore, a different selection of “adjusting” variables in a model (in addition to age and gender) did not significantly alter the aOR and “p” values of either anthropometric measurement. In a sensitivity analysis (Supplementary Table 3), conducted by implementing all socio-demographic and medical history data in a model, all three anthropometric measurements remained significant predictors of primary end-points, with % BF having an increase of aOR at the expense of a wider confidence interval range. In addition to anthropometric parameters, the model showed a significant predictive value of the Charlson comorbidity index for mortality and female sex for ICU admission.

Literature data agree that obesity, defined by BMI, is a significant predictor of disease severity (OR 1.47–5.47) (3–6, 28, 29), need for intensive care unit (OR 1.29–5.49) (3, 5, 6), and invasive mechanical ventilation (OR 1.2–6.01) (3, 5, 6, 17). However, the results regarding mortality are still inconsistent. Although some studies advocate obesity, defined by BMI, as a significant predictor of mortality, with OR ranging from 1.04 to 4.4, or even higher (1, 3, 4, 26, 30–32), others failed to show statistical significance or even showed negative predictive values (3, 5, 6, 16, 33, 34).

A relatively wide range of OR values in these studies, as well as lack of statistical significance for mortality, could be explained by different BMI cut-offs, sample size, diversity of study population (regarding diverse socio-demographic and comorbidity characteristics of the cohort, as well as different COVID-19 severity among patients), the predominance of different SARS-CoV-2 mutation variants, and other. Also, it is important to point out that all cited studies used BMI as the only measurement for defining obesity, possibly leading to misinterpretation of body composition by neglecting total body and visceral fat, especially in older and more comorbid patients (2). This is important because the majority of mechanisms by which obesity adversely affects the course of SARS-CoV-2 infection (chronic inflammation and immune response dysregulation, endothelial dysfunction and increased thrombogenic potential, endocrine dysfunction, etc.) are mostly effects of the adipose tissue (2, 4, 9). In addition, several studies in which abdominal adipose tissue had been evaluated using CT scan emphasized the importance of visceral adipose tissue on COVID-19 severity and mortality (10, 20–23). One of the pathophysiological explanations of this phenomenon lies in the fact that visceral adipose tissue, compared to subcutaneous, secretes 2–3 times higher concentrations of interleukin 6 (35), which is associated with the development of severe forms and fatal outcomes for patients with COVID-19 (1, 7). In our cohort, patients with excessive VF had significantly higher serum levels of CRP (*p* = 0.014) and IL-6 (*p* = 0.028) on admission compared to those with normal VF levels, possibly suggesting higher inflammation grade. We also note that patients with very high % BF had significantly higher values of LDH (*p* = 0.024), another notable COVID-19 predictor (1, 7), while no statistically significant relationship was found between BMI and any proinflammatory marker on admission. Finally, despite a good agreement between BMI and % BF in defining obesity, 24.5% of patients with very high % BF values were categorized as non-obese according to BMI. We accentuate the high incidence of mortality and ICU admission in this group of patients (24.8 and 55.6%, respectively).

All stated mechanisms could explain, at least partially, why BMI lacked statistical significance in terms of predicting mortality of patients with COVID-19 in cited studies. Also, stated mechanisms could explain why BIA measurements, both % BF and VF, had higher OR in predicting each primary end-point compared to BMI. Due to the relatively small sample size and other study limitations, perhaps the exact OR values for anthropometric measurements could not be generalized, particularly in terms of mortality. However, the results are suggestive of a link between obesity and COVID-19 severity and mortality.

## Conclusion

Obesity is a globally raging pandemic that is, in addition to many other comorbidities and all-cause mortality, a significant predictor of COVID-19 severity and death. For that reason, intensive obesity prevention campaigns and programs should be one of the main focuses of healthcare systems worldwide.

Bioelectrical impedance analysis measurements could be a helpful tool in predicting COVID-19 severity and mortality on admission.

By having insight into the total body and visceral fat distribution, BIA measurements (both % BF and VF) were stronger predictors of each primary end-point (mortality and ICU admission) compared to BMI.

## Study Limitations

Our study had several limitations. First, COVID-19, like infection and inflammation, can impact body composition. To minimize that effect, we have measured patients in the initial days of hospitalization. Second, a substantial number of patients were excluded from the study, because of their inability to undergo BIA assessment (such as poor general condition, dementia, and lack of limbs). Third, we have included a relatively small number of patients for the generalization of the results.

Finally, although BIA measurements have satisfactory insight into total body fat and fat-free mass and are widely used for body composition assessment in the general population, this method has difficulty distinguishing visceral from abdominal fat, for which CT and MRI remain the gold standard (36, 37). Due to stated study limitations and presented results, the authors suggest and encourage continuing research on this issue.

## Data Availability Statement

The raw data supporting the conclusions of this article will be made available by the authors, without undue reservation.

## Ethics Statement

The studies involving human participants were reviewed and approved by the University Clinical Center Kragujevac, Kragujevac, Serbia. The patients/participants provided their written informed consent to participate in this study.

## Author Contributions

DS, VZ, MiP, VM, and IC contributed to conception and design of the study. VZ, MaP, IC, and VM ensured quality of the research and performed final review of the manuscript. DS and MiP organized the database. SM and NZ performed the statistical analysis and contributed to presentation of results. DT, AD, MM, DB, AA, and AJ contributed to data collection and helped in writing chapters of the manuscript. DS and MiP wrote the first draft of the manuscript. All authors contributed to manuscript revision, read, and approved the submitted version.

## Conflict of Interest

The authors declare that the research was conducted in the absence of any commercial or financial relationships that could be construed as a potential conflict of interest.

## Publisher’s Note

All claims expressed in this article are solely those of the authors and do not necessarily represent those of their affiliated organizations, or those of the publisher, the editors and the reviewers. Any product that may be evaluated in this article, or claim that may be made by its manufacturer, is not guaranteed or endorsed by the publisher.
