# Supplementary material for: The Role of Bioelectrical Impedance Analysis in Predicting COVID-19 Outcome
Source: Front Nutr. 2022 Jul 11;9:906659. doi: 10.3389/fnut.2022.906659 (PMC9310439; doi:10.3389/fnut.2022.906659)
Supplement: Supplementary file 1 [file Table_1.DOCX]

Supplementary Table 1. Median values of inflammatory biomarkers on hospital admission, presented by different anthropometric measurement categories.

| Anthropo-metric measurement | Anthropo-metric categories | Inflammatory biomarkers [median (IQR)] | | | | | |
| --- | --- | --- | --- | --- | --- | --- | --- |
|  |  | Fibrinogen | LDH | Ferritin | CRP | PCT | IL-6 |
| BMI | ≥ 30 kg/m^2^ | 6.13  (2.23) | 780.0 (343.0) | 877.0  (793.0) | 100.0  (103.0) | 0.142 (0.130) | 67.0 (121) |
|  | < 30 kg/m^2^ | 6.59  (2.08) | 746.0  (422.0) | 796.0 (775.0) | 97.0  (94.0) | 0.117 (0.195) | 52.8  (86.6) |
| %BF | Obese | 6.42  (2.15) | 793.5  (325.0) * | 899.0  (717.0) | 102.5 (103.0) | 0.087 (0.152) | 65.4 (116.7) |
|  | Non-obese | 6.50  (2.16) | 701.0  (453.0) | 762.0  (833.0) | 96.25 (90.25) | 0.122 (0.197) | 50.8  (86.6) |
| VF | Very high | 6.61  (2.13) | 780.0  (366.0) | 945.5 (655.25) | 116.2 (108.3) * | 0.117 (0.201) | 88.0  (113.6) * |
|  | Normal/High | 6.27  (2.11) | 727.0  (426.0) | 735.5 (819.5) | 88.8  (94.5) | 0.090 (0.145) | 50.4  (80.0) |

Abbreviations: %BF - Body fat percentage; BMI - Body mass index; CRP - C-reactive protein; IL-6 - Interleukin-6; IQR - Interquartile range; LDH - lactate-dehydrogenase; PCT - Procalcitonine; VF - Visceral fat.

* Statistical significance with "p" values < 0.05, using the Mann-Whitney U test.
