# Supplementary material for: The Role of Bioelectrical Impedance Analysis in Predicting COVID-19 Outcome
Source: Front Nutr. 2022 Jul 11;9:906659. doi: 10.3389/fnut.2022.906659 (PMC9310439; doi:10.3389/fnut.2022.906659)
Supplement: Supplementary file 2 [file Table_2.DOCX]

Supplementary Table 2. Crude OR (with 95% CI and "p" values) for socio-demographic characteristics and comorbidities in regards to the primary end-points occurrence (mortality, ICU admission, and either primary end-point)

| **Characteristics** | | **Mortality** | | **ICU admission** | | **Either primary end-point** | |
| --- | --- | --- | --- | --- | --- | --- | --- |
|  |  | Crude OR (95% CI) | P-Value | Crude OR (95% CI) | P-Value | Crude OR (95% CI) | P-Value |
| **Age** | 10 years increase | 1.407  (0.998-1.993) | 0.052 | **0.780**  (0.614-0.991) | 0.042 | 0.827  (0.653-1.046) | 0.113 |
| **Sex** | Male | 1 |  | 1 |  | 1 |  |
|  | Female | 1.264  (0.610-2.620) | 0.529 | **2.006**  (1.124-3.579) | 0.018 | 1.741  (0.982-3.085) | 0.058 |
| **Arterial hypertension** | No | 1 |  | 1 |  | 1 |  |
|  | Yes | 1.300  (0.589-2.871) | 0.516 | 0.733  (0.404-1.328) | 0.305 | 0.758  (0.421-1.365) | 0.356 |
| **Diabetes mellitus** | No | 1 |  | 1 |  | 1 |  |
|  | Yes | 1.545  (0.714-3.444) | 0.269 | 1.246  (0.661-2.349) | 0.496 | 1.235  (0.659-2.314) | 0.509 |
| **Chronic kidney disease (grade III-V)** | No | 1 |  | 1 |  | 1 |  |
|  | Yes | 2.800  (0.976-8.035) | 0.056 | 0.534  (0.169-1.685) | 0.285 | 1.164  (0.432-4.136) | 0.764 |
| **Atrial fibrillation** | No | 1 |  | 1 |  | 1 |  |
|  | Yes | 0.339  (0.043-2.669) | 0.303 | 0.128  (0.016-0.993) | 0.049 | 0.258  (0.057-1.177) | 0.080 |
| **Malignancy** | No | 1 |  | 1 |  | 1 |  |
|  | Yes | 0.904  (0.192-4.262) | 0.898 | 0.570  (0.152-2.139) | 0.405 | 0.791  (0.236-2.660) | 0.705 |
| **Previous myocardial infarction** | No | 1 |  | 1 |  | 1 |  |
|  | Yes | 0.614  (0.074-5.069) | 0.651 | 0.979  (0.238-4.030) | 0.976 | 0.899  (0.218-3.698) | 0.882 |
| **Obstructive lung disease** | No | 1 |  | 1 |  | 1 |  |
|  | Yes | 4.000  (0.855-18.704) | 0.078 | 0.777  (0.147-4.108) | 0.767 | 1.368  (0.298-6.278) | 0.687 |
| **Neurological condition** | No | 1 |  | 1 |  | 1 |  |
|  | Yes | 1.000  (0.113-8.824) | 0.983 | 0.979  (0.175-5.474) | 0.981 | 0.900  (0.161-5.030) | 0.904 |
| **CCI** | 1 point increase | **1.372**  (1.147-1.641) | 0.001 | 0.924  (0.795-1.074) | 0.304 | 1.008  (0.873-1.163) | 0.917 |
| **Days from disease onset** | 1 day increase | **0.876**  (0.789-0.972) | 0.013 | 0.940  (0.874-1.012) | 0.102 | **0.923**  (0.857-0.994) | 0.034 |
| **BMI** | Non-obese | 1 |  | 1 |  | 1 |  |
|  | Obese | 1.930  (0.939-3.971) | 0.075 | **3.775**  (2.087-6.827) | 0.000 | **3.187**  (1.784-5.693) | 0.000 |
| **BF%** | Non-obese | 1 |  | 1 |  | 1 |  |
|  | Obese | **2.540**  (1.179-5.470) | 0.017 | **7.602**  (3.868-14.960) | 0.000 | **6.282**  (3.312-11.918) | 0.000 |
| **VF** | Normal/High | 1 |  | 1 |  | 1 |  |
|  | Very high | **3.066**  (1.466-6.411) | 0.003 | **2.364**  (1.325-4.219) | 0.004 | **2.414**  (1.360-4.284) | 0.003 |

Abbreviations: %BF - Body fat percentage; BMI - Body mass index; CCI - Charlson comorbidity index; CI - Confidence interval; Either primary end-point - fatal outcome and/or ICU admission; Neurological condition: history of stroke, brain tumor, or malformation, vascular disease, dementia of any etiology, etc.; Obstructive lung disease – the presence of either chronic obstructive lung disease or bronchial asthma; OR - odds ratio; VF - Visceral fat.
