# Supplementary material for: The Role of Bioelectrical Impedance Analysis in Predicting COVID-19 Outcome
Source: Front Nutr. 2022 Jul 11;9:906659. doi: 10.3389/fnut.2022.906659 (PMC9310439; doi:10.3389/fnut.2022.906659)
Supplement: Supplementary file 3 [file Table_3.DOCX]

Supplementary Table 3. Sensitivity analysis comparing aOR (with 95% CI and "p" values) for anthropometric measurements in regards to primary end-points occurrence presented in Table 1 with OR adjusted for all available socio-demographic and medical history data.

| Anthropometric measurement method | Body composition categories | End-point occurrence  (%) | Adjusted OR (95% CI) * | *p* | Fully adjusted OR  (95% CI) ** | *p* |
| --- | --- | --- | --- | --- | --- | --- |
| **Mortality** | | | | | | |
| BMI | Non-obese | 13% | 1 |  | 1 |  |
|  | Obese | 22.4% | **2.387** (1.067-5.337) | 0.034 | **2.878** (1.213-2.961) | 0.005 |
| %BF | Non-obese | 10.4% | 1 |  | 1 |  |
|  | Obese | 22.7% | **3.353** (1.471-6.642) | 0.004 | **4.525** (1.693-12.090) | 0.003 |
| VF | Normal/High | 10.5% | 1 |  | 1 |  |
|  | Very high | 22.6% | **3.050** (1.407-6.609) | 0.005 | **3.424** (1.435-8.172) | 0.006 |
| **ICU admission** | | | | | | |
| BMI | Non-obese | 22.1% | 1 |  | 1 |  |
|  | Obese | 51.8% | **3.113** (1.663-5.825) | <0.001 | **2.865** (1.450-5.663) | 0.002 |
| %BF | Non-obese | 13.2% | 1 |  | 1 |  |
|  | Obese | 53.6% | **7.141** (3.538-14.413) | <0.001 | **7.550** (3.487-16.347) | <0.001 |
| VF | Normal/High | 26.3% | 1 |  | 1 |  |
|  | Very high | 56.8% | **3.424** (1.781-6.581) | <0.001 | **3.287** (1.649-6.553) | 0.001 |
| **Either primary end-point** | | | | | | |
| BMI | Non-obese | 25.2% | 1 |  | 1 |  |
|  | Obese | 51.8% | **2.769** (1.495-5.125) | 0.001 | **2.885** (1.464-5.682) | 0.002 |
| %BF | Non-obese | 16% | 1 |  | 1 |  |
|  | Obese | 54.5% | **6.085** (3.121-11.862) | <0.001 | **7.643** (3.543-16.488) | <0.001 |
| VF | Normal/High | 27.8% | 1 |  | 1 |  |
|  | Very high | 48.2% | **3.208** (1.705-6.035) | <0.001 | **3.338** (1.697-6.567) | <0.001 |

Abbreviations: %BF - Body fat percentage; BMI - Body mass index; CI - Confidence interval; ICU - Intensive care unit; OR - odds ratio; VF - Visceral fat.

** OR was adjusted for age, sex, days from disease onset, and diabetes mellitus.*
*** OR was adjusted for age, sex, arterial hypertension, diabetes mellitus, chronic kidney disease, atrial fibrillation, malignancy, previous myocardial infarction, obstructive lung disease, neurological condition, Charlson comorbidity index, and days from disease onset.*
